# Predictive and preventive strategies to advance the treatments of cardiovascular and cerebrovascular diseases: the Ukrainian context

**DOI:** 10.1186/1878-5085-3-12

**Published:** 2012-10-19

**Authors:** Ulyana B Lushchyk, Viktor V Novytskyy, Igor P Babii, Nadiya G Lushchyk, Lyudmyla S Riabets

**Affiliations:** 1 Research Center Veritas, 31 Obolonska Str., of. 9, Kyiv 04071, Ukraine; 2 Clinic Victoria Veritas, 4 Williams Str., Kyiv, 03191, Ukraine; 3 Center for Innovative Medical Technologies Veritas IT Med, 4 Williams Str., Kyiv, 03191, Ukraine

**Keywords:** Cardiovascular diseases, Hemodynamics, Capillaroscopy, Predictive diagnostics, Preventive strategies, Personalized medicine

## Abstract

Despite great efforts in treatments of cardiovascular diseases, the field requires innovative strategies because of high rates of morbidity, mortality and disability, indicating evident deficits in predictive vascular diagnosis and individualized treatment approaches. Talking about the vascular system, currently, physicians are not provided with integrated medical approaches to diagnose and treat vascular diseases. Only an individual global approach to the analysis of all segments in the vascular system of a patient allows finding the optimal way for vascular disease treatment. As for the existing methodology, there is a dominance of static methods such as X-ray contrast angiography and magnetic resonance imaging in angiomode. Taking into account the world experience, this article deals with innovative strategies, aiming at predictive diagnosis in vascular system, personalization of the biomedical treatment approaches, and targeted prevention of individual patient cohorts. Clinical examples illustrate the advances in corresponding healthcare sectors. Recommendations are provided to promote the field.

## Review

### Cardiovascular disease is an actual problem in many countries: results of marketing-standardized approach to problems of a specific patient

The background level of functioning of nearly all organs and systems is important for providing blood pressure in the cardiovascular system. Today, nobody can deny the fact that any organ dysfunction can be caused by decrease or sudden stop of the blood supply. A theory, which exists in medicine years long, stating that any metabolic disturbance is the primary pathogenetic factor of cardiovascular diseases (CVDs) and disorders in blood supply level is the secondary one is losing its significance now [1-13].

CVDs are an actual problem nowadays because of high rates of morbidity, mortality, and disability, indicating the low efficiency of applied methods for vascular diagnosis and treatment [7,10,11,14-17]. Today, occupying first place by spreading cardiovascular diseases cause more than half of all deaths and one-third of disability, mainly due to uncompensated cardiovascular conditions—heart attacks and strokes (according to statistics from WHO and Ministry of Health of Ukraine).

Three-fourths of the population is suffering from cardiovascular pathologies in Ukraine alone, and it causes death in 62.5% of cases; that is much higher than in developed countries. Recently the spreading of ischemic heart disease has increased in Ukraine from 10 thousands to more than 20 thousands per 100 thousands of the population, and more than 5 million patients with hypertonic disease are registered in Ukraine [10,11].

When we analyzed the statistics in Ukraine, Europe, and the world [8,17], we observed the following tendency: 20 years ago, people older than 40–50 years had CVDs, while today the age of such patients is older than 30 years. Lately, there is a negative tendency in Ukraine: strokes began to be more frequently observed in teenagers and children of preschool and junior school age [6,10].

To the greatest regret, today's statistics of Ukraine and other countries of the world represent mainly negative tendencies except for positive Japanese experience [8,16], namely cardiovascular diseases are an urgent medical and social problem nowadays because of high rates of morbidity, mortality and disability, indicating the low efficiency of applied methods for vascular diagnosis and treatment [4-7,9,18,19].

For the last 10 years, only single works have described studies of arteriovenous cerebral balance as a sign of hydrodynamic and volumetric disbalance in the interdependent arterial and venous links of the brain vascular system [9,17,20]. During the last 2 decades, many scientific investigations have been aimed at studying problems of diagnostics and pathogenesis of cardiovascular diseases; there are methods of *in vivo* noninvasive examination of the vascular system on macro- and micro-levels. There is a tendency to create vascular pathology departments in medical establishments. Physicians pay more attention to the combined vascular cardioneurological pathology [4,5,8,14,17-19,21,22].

### Current problems in diagnostics and treatments in Ukraine: can a personalization of the approach be possible?

Despite of considerable efforts of scientists, there is no tendency to decrease morbidity and mortality indexes of cardiovascular diseases today. In fact, the world has not enough efficient technologies for preventive examination of the cardiovascular system (CVS) [1-3,10-12,15,20,23-28]. They must not be for palliative adaptation to the sickly state, but for restoration of the system to the level of autoregulation and self-control. Some important aspects in evidence-based medicine have not been included in basic and applied research. Let us name the most important:

1. Generally local structural examinations of separate CVS segments prevail in CVD research. Local CVS examination does not consider interconnections between dynamics of segments and general dynamics of the vascular system on various regional levels.

2. There is no systemic approach to the examination of CVS as an entire system of vascular ‘hemo-supply’ with multiple intersystemic connections.

3. A role of arterial and venous dampers is ignored for blood redistribution in various regional reservoirs.

4. The venous system is not examined enough as it is considered to be in a shadow and less accessible for life-time functional examination.

5. Current diagnostic and treatment measures are not sensitive enough for early disorders in CVS functioning.

6. One-sided CVS examination. There is a gap between local medical examination and a global approach under mathematical modeling of CVS according to cybernetics because of the lack of local indicators for the vascular system condition. One cannot make global conclusions about the functioning of the entire system basing on one CVS parameter. Such an approach to the CVS investigation is too expensive, thus causing ‘rejuvenation’ and progression of CVDs.

7. Lack of a single approach *in vivo* to blood as a biological and biochemical non-Newton liquid causes physicians to be mistaken about the properties of blood—it is perceived as an ordinary liquid.

8. Usage of absolute values as a statement of incorrect functioning of the system not taking into account parameters of reactivity and adaptation of CVS in conditions of internal homeostatic imbalance and changes of environmental parameters (meteorological factors), and neglecting integral parameters when estimating CVS functioning causes a principally wrong static (but not dynamic) approach in analyzing the functioning of the dynamic blood circulation system with many variable, in the certain range, one moment hemodynamic indexes in various CVS segments.

However, it is only a top of an iceberg called ‘pathology of the vascular bed’ because today, the situation of venous stagnation in organs of the small pelvis, hypertension in pregnants, and vascular anomalies in new-born is out-of-control, and the only thing applied to fight hypertension is tonometry.

This shows that the generally accepted approaches predetermine small efficiency of diagnostic and therapeutic procedures because of poor sensitivity of the applied diagnostic methods for early disorders in CVS functioning and lack of effective technologies of these methods' application.

Even in the theory, not to mention in practice, physicians do not have a single integrated approach to the vascular system. As for existing methods for diagnosing circulatory system diseases, we can talk about the dominance of static methods such as X-ray contrast angiography and magnetic resonance imaging in angiomode [20].

Ability to detect atherosclerotic plaques and thromboembolus applying the newest diagnostic methods provoked the creation of new fields in medicine—angiosurgery and cardiosurgery with allegedly radical approach—which found the cause of and managed vascular decompensation. Everything seems to be acceptable, but why do some post-operated patients often have temporary improvement and relapses often occur?

The catamnesis shows deeper disorders in the whole CVS as a complex system of interconnected tubes with different caliber and specific features of their walls and biophysical blood properties that can be called as a liquid only in theory.

Any clinical result in medical practice is considered as positive when a patient has stable positive angioneurological dynamics during treatment and durable positive disease catamnesis after treatment completion. In a global scale, these criteria of treatment efficiency result in the reduction of morbidity indexes, death rate, and disability caused by cardiovascular diseases.

Current new ideologies in the application of principles of evidence-based medicine in the medical management of CVDs and the potential of modern medical technique allow the realization of the predictive IT program of the individually oriented approaches for early detection, prevention of vascular crises, and treatment of CV pathology to be seen in a new light. According to the CVS treatment tactics, the approaches must not for palliative adaptation to a sick condition but for restoration of the system to the level of autoregulation and self-control.

#### Recommendations

Patients' recovery and decreased disability and mortality are the primary objectives of medicine. In the last decade, the business-marketing approach in medicine offered by pharmaceutical and medical–technical industries has converted medical industry into a source of super profits for pharmaceutical and medical–technical globalized businesses by means of the formation of treatment standards. The negative result of such strategy has leveled the primary objective of the medicine and was shown to be associated with the increase of morbidity, death rate, and disability of CVD patients.

As a result, people lose treatment methods and doctors' art elaborated for years as the standardized approach to ‘sales of medications’ and ‘robotization’ of organs does not take into account the features of the pathogeny of the integral system of the human organism and the course of combination of diseases in a specific patient.

The current level of examination of the cardiovascular system requires new analytical approaches for the prevention of CVDs, insults and infarctions in people of different age, and diminishing of disability and mortality of vascular critical states. In this connection, principles of predictive, preventive, and personalized (PPP) medicine become principally actual [15].

### Ukrainian integrated medical technologies for CVS examination

Lately, there are positive tendencies of medical cluster creation in Ukraine [9,22]. Combination of innovative projects within the framework of scientific centers, clinics, and centers for the development of new medical technologies is interesting enough and perspective [21,29]. Combining efforts of the specialists' team for the creation of innovative approaches for diagnostics and treatment of cardiovascular diseases is an example of the cluster, which has been functioning since 1996 and engaged in profound scientific investigations and modeling of any vascular problems. The innovative medical technologies developed by the cluster enable considerably to reduce morbidity indexes of cardiovascular pathologies in the population. Beginning with a methodology of ultrasound dopplerography of arteries and veins of the brain [30], they have created unique developments in the field of angioneurology with the investigation of angioarchitectonics, arteriovenous balance, microcirculation bed, and the formation of some logical approaches to controlled changes in hemodynamics of the regional reservoirs [25-28].

The model of symbiosis of the scientific and practical medical establishments allow for the improvement of generally accepted technologies for diagnostics and treatment of cardiovascular and cerebrovascular diseases in 3,424 patients for 16 years. A theory of vascular pipeline with moving blood as non-Newtonian liquid lies in the basis of these innovative studies [9,28-31]. The theory allows estimating a structure and function of vascular blood pipeline from positions of unity of arterial, venous, and capillary links in different regional reservoirs. The poly-vector approaches to the estimation of CVS functioning enable to diagnose the state of arteriovenous balance, arteriolar–venular balance, and their displacement, and estimate a key nosotropic factor in pathological reconstructions of the vascular bed: atherosclerosis, arteritis, aneurysm, sinuosity, tortuosity, hypoplasia, phlebectasia, congenital anomalies of angioarchitectonical formation, thrombosis, stenotic-occlusive pathology, etc.

Current diagnostic methods of CVS like CТ, МRТ in angiomode, US scanning have static nature and mainly they insufficiently estimate CVS functioning. ECG represents mainly the bioelectric state of myocardium and, being a diagnostic method, it does not allow estimating sufficient functioning of the heart as a pump for vascular blood pipeline. EchoCG estimates the pumping function of the myocardium only from a position of discharge fraction not taking into account hemodynamic features like plasticity, adaptogenity, and intravascular resistances of the whole CVS. Therefore, all these methods give insufficient information for analytical estimation of CVS functioning in blood supply for organs and systems [9,13,22,25-27,29-32].

The approach enables to detect the combination of basic pathogenetic factors that initiate a cascade of pathological alterations in the structure and function of the vascular pipeline [10,33]. Indexes of sufficient and adequate blood supply; blood pressure; renewal of balance between elasticity and tone of the vascular wall, and intravascular pressure and distal resistance; a level of hydrodynamic conflict between the vessel and surrounding tissues are the most substantial criteria, which represent some positive dynamics in the restoration of the blood supply for the organ. Therefore, medicinal treatment included the exact analysis of these hemodynamic parameters for the estimation of treatment dynamics.

Analyzing the positive results of treatment and marking the stability of retaining the results for 6 months to 2 years, we gradually have created our own medical technologies for personalized objectification of a vascular pathology for prophylaxis and prevention of critical states. More detailed research of peculiar functioning of the cardiovascular system as a complex vascular pipeline in virtually healthy patients with minimum complaints about worsening of their work ability and periodic discomfort during weather changes suggested us an idea concerning the necessity of revision and correction of the generally accepted conception of segmental research and treatment of the arterial pathology.

Such approach to complex CVS examination has motivated the necessity of deeper study of the many-sided aspects of the living pipeline functioning, namely, the vascular wall functioning, changes of elasticity and tonus in arterial and venous vessels, internal vascular peripheral resistances, intravascular transversal and longitudinal pressures, and arteriovenous balance.

For completion of absolute understanding of the whole cardiovascular system as an integral entity, an urgent need in the microcirculatory research occurred because microcirculation is a system of complicated interconnections of arteriolar and venular segments of capillaries and the most distant CVS segment from the heart.

We got positive results of integrated treatment of differently aged CVD patients applying the method of the personalized intensive medicinal correction of the vascular pathology [13,25,30], which have showed an increase of treatment efficiency of 2–5 times with reduction of treatment period to 1–2 months.

The treatment effectiveness is the absence of vascular crises, meteoropathy signs and subjective discomfort, and work-ability renewal and remaining stable for 3–6 months accompanied by the stable state of blood supply for the organism nearly without any daily background medicinal therapy during an intercourse period.

### Strategic predictive, preventive, and personalized approaches to the development and creation of analytical evidence-based medical technologies for CVS examination and correction

The organism is considered to be a controlled system, to peculiarities of hydrohemodynamic laws *in vivo* for providing functioning of interdependent segments of the closed CVS: *heart → major arteries → peripheral arteries → arterioles → capillaries → venules → peripheral veins → major veins → heart* that is why one-moment examination of CVS requires quite new technological approaches with the detection of polyvector characteristics of all levels and concretization of an injured area and influence of the area on the functioning of the whole system [9].

The experience of instrumental diagnosis of disorders in cardiovascular system with ultrasound dopplerography, ultrasound scanning and smart capillaroscopy, MRT in angiomode, and effective clinical results of integrated treatment of CVD patients at all ages gradually formed a view about urgent need in the integrated approach to diagnosis and correction of changes in CVS including the following principles of evidence-based medicine:

• predictive testing of the treatment scheme effectiveness by means of individual acute medical tests with possible correction with the help of feed-back options;

• an analytical approach to profound interpretation of pathological or sanogenic changes in the vascular system, but not just statement of absolute parameters of blood flow;

• prevention: fixing results by improving the function of CVS into physiologically stable type, with the restoration of the stable balance in the dynamic system;

• early preclinical diagnostics of vascular changes enable to detect vascular pathological alterations at the initial stages and to avoid CVS disbalance to the critical level. This is a very important preventive approach for the prophylaxis of stroke and heart attack as displays of CVS decompensation at different levels.

• personalization: individual approach to the simultaneous integrated CVS examination in a particular patient; individual control of the effective treatment, owing to monitoring by methods of the evidence-based medicine.

We consider that the classic standardized approaches to the treatment of a disease, but not a certain patient, must become a thing of the past. Today's level of evidence-based medicine allows and requires an individual approach to every patient which, with particular doctor's knowledge, enables to get some substantial positive results in treatment and to stabilize the situation.

Thanks to the methodology for examination and clinical interpretation of hemodynamic reconstructions developed by our specialists, the ultrasound dopplerography has come up to a level of modern innovative medical technology that enables considerably to improve the state of the cardiovascular system functioning and reasonably to correct it, taking into account detected pathologies. Only an inexperienced doctor affords to ignore the dopplerography results of the main and peripheral vessels [34]. It is necessary to analytically estimate the results of separate local instrumental examinations with their generalization within the limits of the single vascular system of the organism and analysis of the synchronous functioning of the different regional reservoirs.

The predictive approach to CVD diagnostics and treatment consists the necessity of multivector investigation of the vascular system as an integral pipeline

1. Arterial link

a) Linear circulation rate

b) Vascular lumen

c) Pressure

d) d Tonus

e) Elasticity of a vascular wall

f) Angioarchitectonics

2. Venous link

a) Linear circulation rate

b) Vascular lumen

c) Pressure

d) Tonus

e) Elasticity of a vascular wall

f) Angioarchitectonics

g) Sate of the valvular apparatus

3. Vascular balance

a) Arteriovenous balance

b) Arteriolar–venular balance

4. Microcirculation as the most sensitive link to early signs of any vascular disorders. Very dynamic CVS examination, which takes into account interconnections between the segments, must reach a principally new stage of intellectual processing of results from different local instrumental examinations with general conclusion within the entire vascular system in the organism. Currently, there is an urgent need for predictive struggle with ‘invasion’ of cardiovascular diseases (Figures 1, 2, 3, 4).

**Figure 1:**
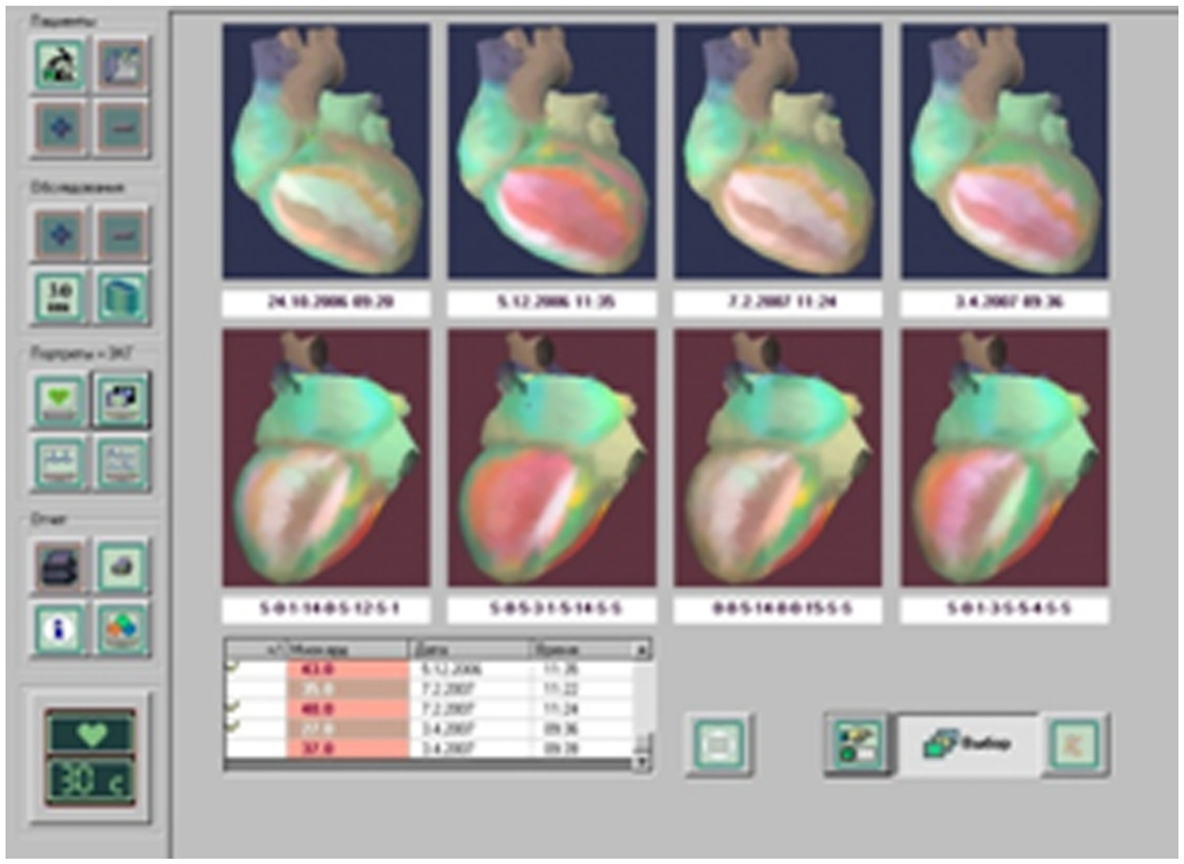
**High trust and understanding of the heart portrait in dynamics by a patient.** The methods of highly sensitive ECG allows the diagnosis and monitoring of early ischemic changes in the myocardium with the correction of vascular blood flow.

**Figure 2:**
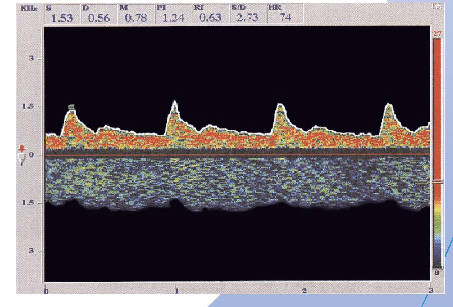
**Clinical interpretation of the functional state of brain arteries and veins through ultrasound dopplerography.** Only methods of clinical interpretation of the functional state of arteries and veins in the brain with the help of ultrasound dopplerography can localize signs of the arteriovenous shunting and define its size and risk of development of deficit in regional blood supply [25-27,30].

**Figure 3:**
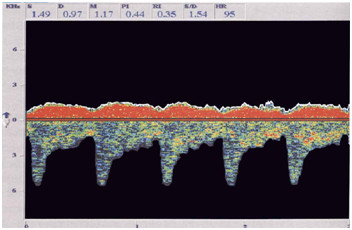
**An example of an examination of the arteriovenous cerebral balance with ultrasound dopplerography**[30]**.** If we do not take into account the degree of violation of this balance, treatment of any vascular pathology in the regional reservoir may be not effective.

**Figure 4:**
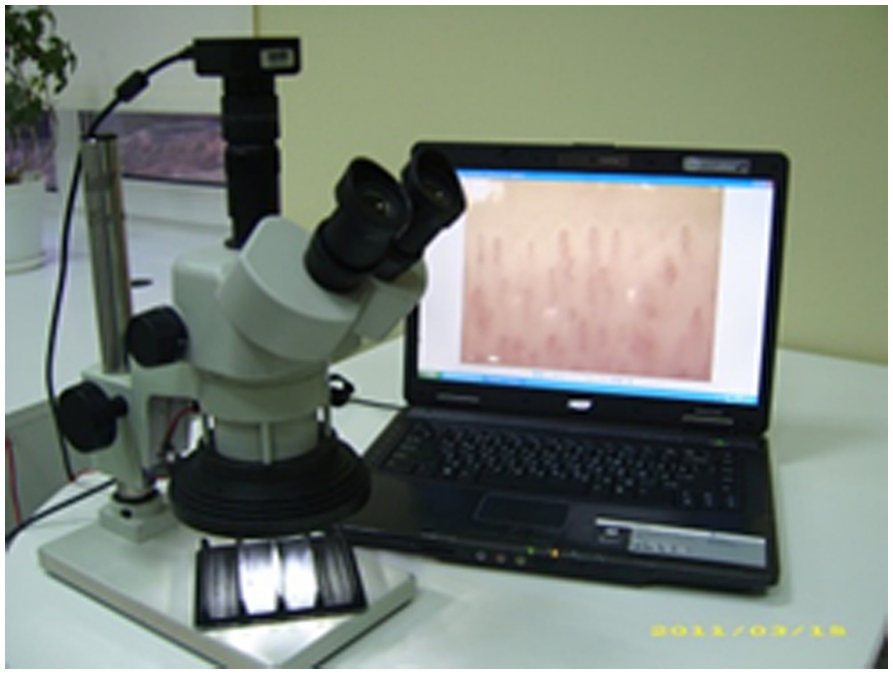
**Equipment in obtaining images.** Due to the obtained images, we can estimate large amount of microcirculatory parameters that enable to estimate the influence of rehabilitation on all stages.

### An algorithm for PPP management of CVD patients

1) Detailed complex diagnostics of the vascular bed enabled to detect nearly 5–8 pathological links in CVS functioning in the sick organism on a stage of early preclinical changes in the organism;

2) Multivector signs of synergy disbalance in the vascular pipeline functioning require one-moment purposeful influence of adequate medicinal remedies individually selected under instrumental control using technologies for prognostication of the end result in the closest month and half-year period;

3) Instrumental monitoring of the dynamics of hemodynamic parameter changes on the principles of evidence-based medicine at intermediate critical periods of sanogenic changes of the vascular bed and timely reaction to pathological meteor tropic reactions, which occur under influence of external factors;

4) The treatment is conducted with the purpose of getting stabilized hemodynamic parameters of the whole system of the vascular plumbing in all regional reservoirs in order to achieve hydro- and hemodynamic balance in the whole system of the vascular plumbing.

Up-to-date highly sensitive devices enable to work both with arterial and venous segments at the same time, and specially developed software calculates what vital deviations are in the patient from the state of arteriovenous balance towards unjustified arterial hyperemia or venous stagnation of one or other degree [34]. Today, the method is necessary not only for functional and ultrasound diagnosticians but also for many medical directions such as reanimation, pediatrics, neonatology, neurology, psychiatry, urology, gynecology and obstetrics, oncology, cardiology, surgery, and neurorehabilitation.

**Figure 5:**
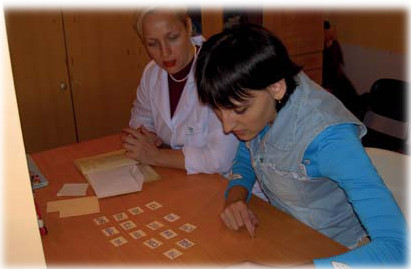
**Interchanging of various neurorehabilitation exercises.** This takes into account a level of blood filling and the brain readiness to loads. Born in 1989. Diagnosis: apallic syndrome, the stage of ‘high’ full consciousness, first degree of disability.

**Figure 6:**
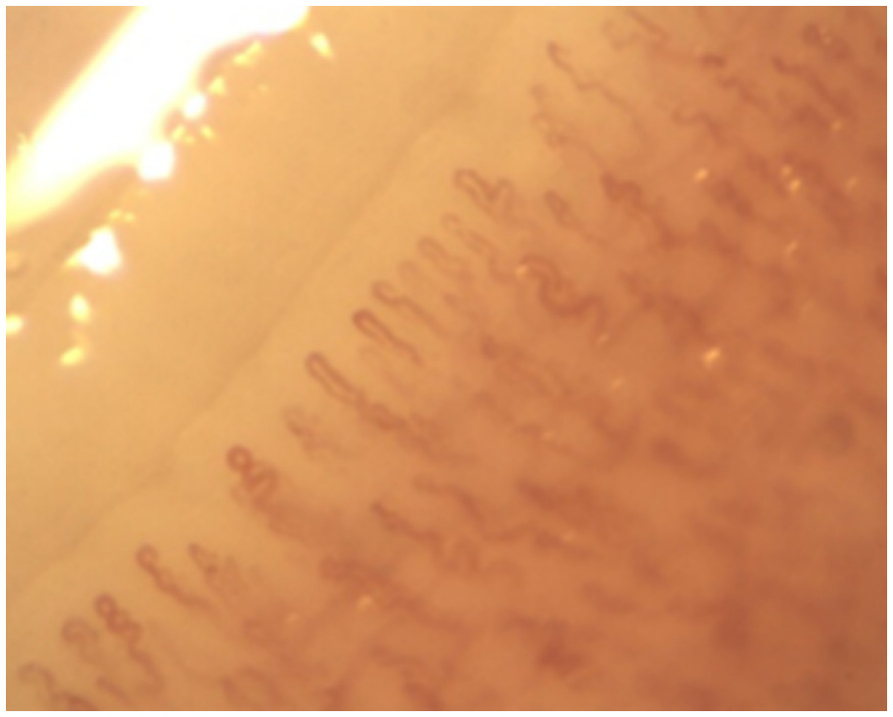
Microcirculatory image.

**Figure 7:**
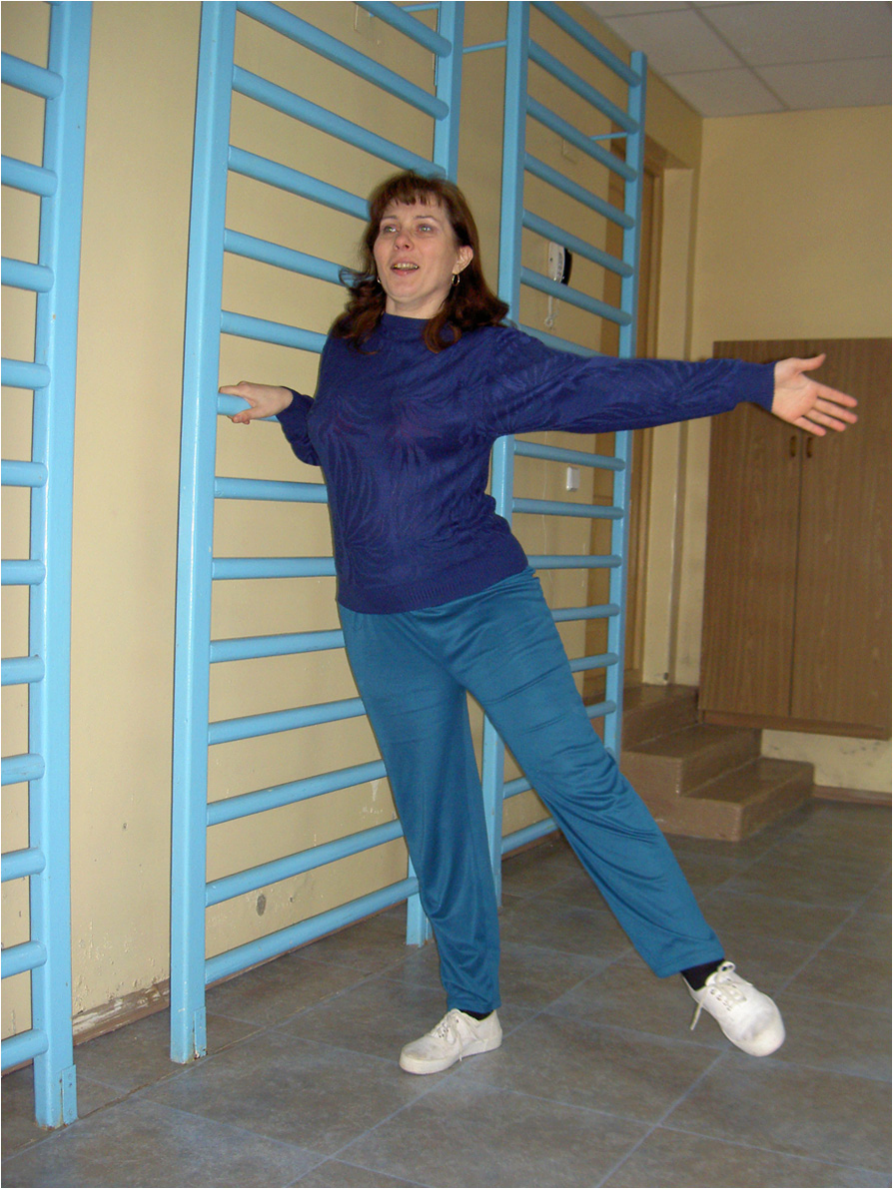
**The six-year rehabilitation program is successfully completed.** Born in 1983. Diagnosis: multiple sclerosis, 1st degree of disability, tied down to the bed.

**Figure 8:**
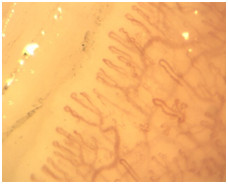
The microcirculatory picture testifies about the necessity of continuation of regular exercises.

**Figure 9:**
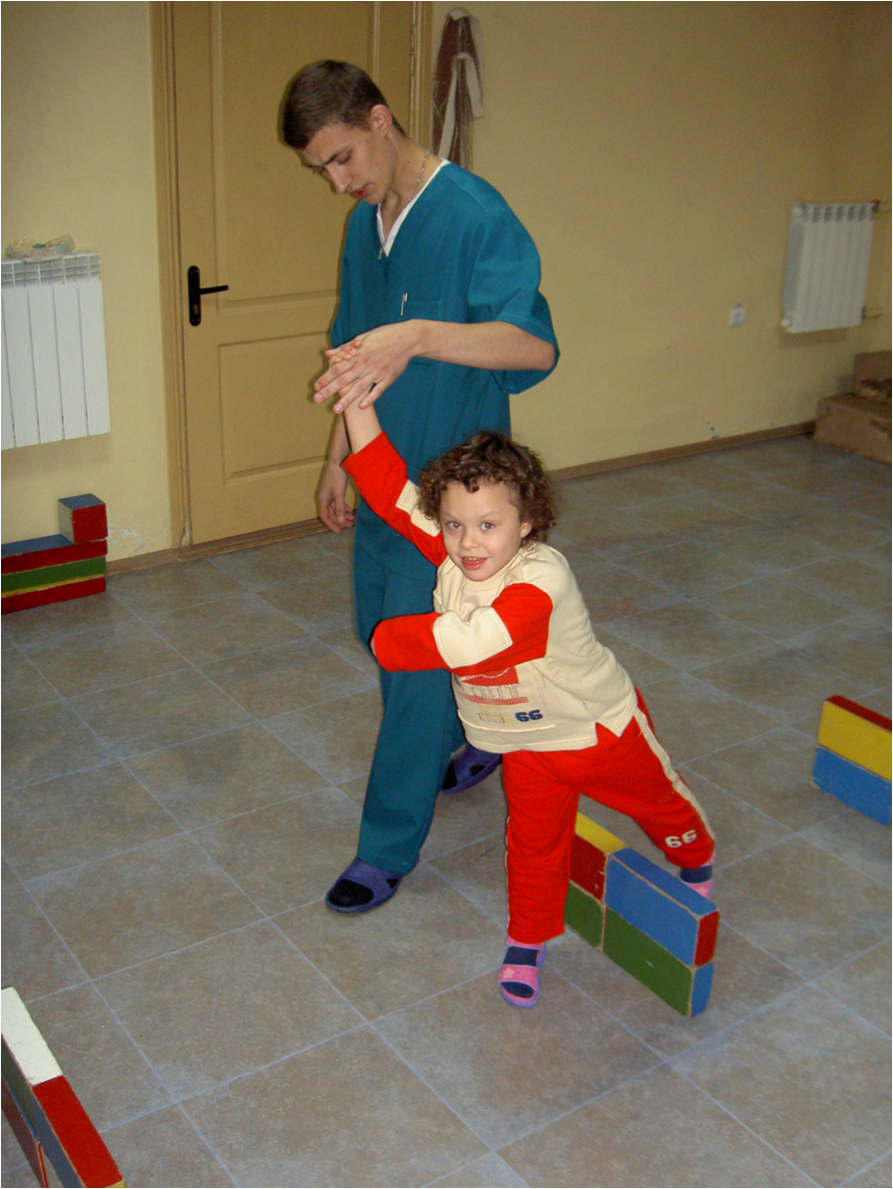
**A stage of preparation to some group activity in a preschool establishment.** This became possible due to the circulation improvement and strengthening of cognitive functions. Born in 2001. Diagnosis: autism, arrest of psycho-motor development.

**Figure 10:**
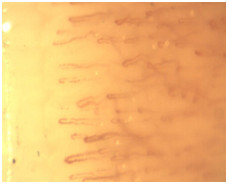
Microcirculatory image.

**Figure 11:**
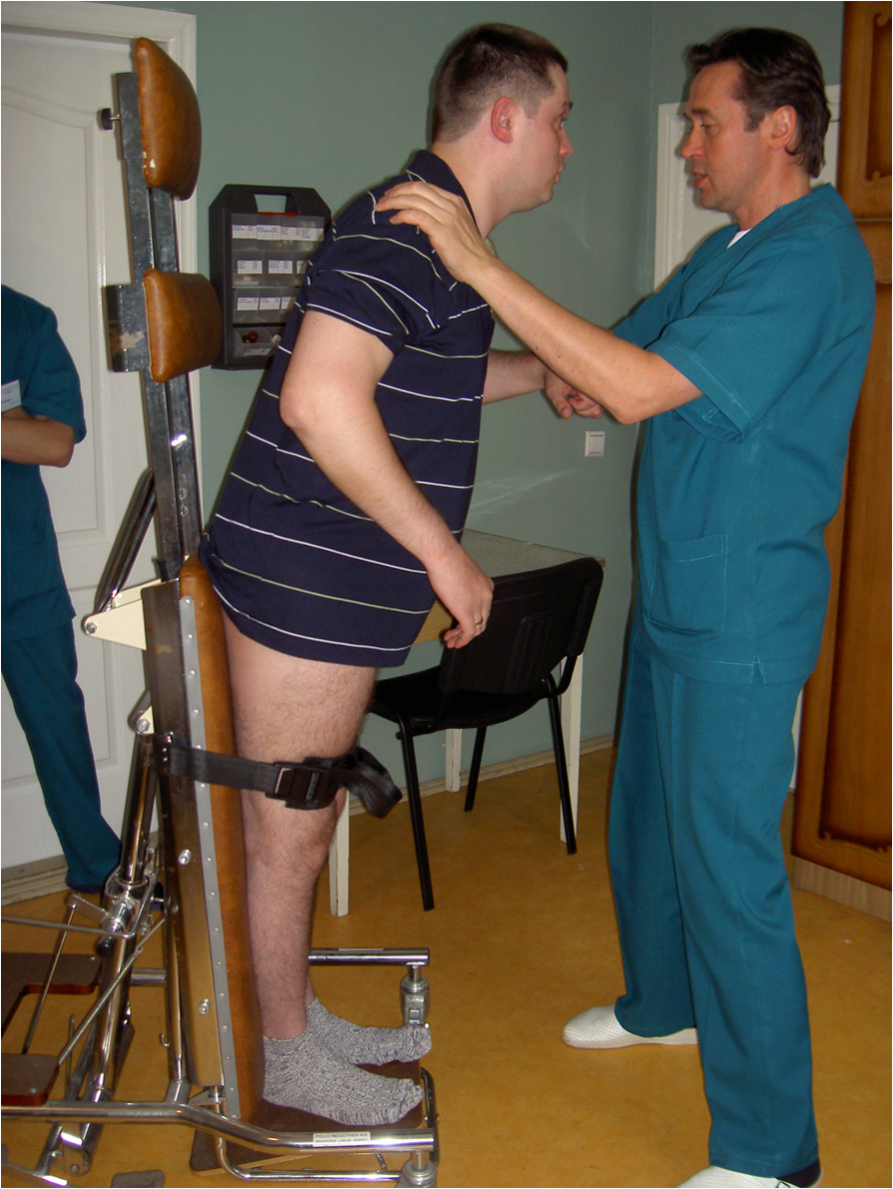
**Psychokinetic therapy.** This helps to restore sanogenic reactions of the vascular system to psychological and physical loadings, and to prepare the vascular system to the body verticalization of the patient after long staying in bed. Born in 1977. Diagnosis: apallic syndrome, stage of ‘large’ (full) consciousness, first degree of disability.

**Figure 12:**
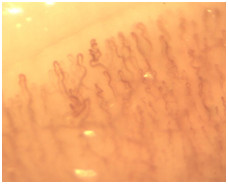
Microcirculatory image.

On the base of our clinic, we apply authorial technologies of examination of arteries and veins in the human organism [30] and we have experienced unique diagnostics and treatment with individual control of the state of vascular channel reconstructions in the organism at CVS dysfunction. Successful results of treatment (Figures 5, 6, 7, 8, 9, 10, 11, 12) of incurable states, such as apallic syndrome, heavy forms of infantile cerebral paralysis, autism, and epilepsy (http://www.inno-health.com.ua, http://www.istyna.kiev.ua, http://www.itmed.com.ua), enabled us to consider background and reserve potential of vascular diagnostics in a new light and to understand logic of pathological and physiological reconstructions in the vascular channel. Indication and verification of control parameters according to the principles of evidence-based medicine enabled to put aside standard approach, to hold an ideology of individuality of changes and variety of combination of pathological links at CVDs, to change attitude toward diagnostics and treatment of CVS as dynamic, but not static system in the human organism.

We have obtained positive results of medicinal CVS correction in every certain case and stable clinical results of recovery and absence of repeated vascular crises during a long time (0.5–1 year), even without application of corresponding medicinal remedies that testify to possible sanogenic reconstruction of the cardiovascular bed when considering the logic of the process.

The personalized approach has showed the effectiveness at different psychoneurological and cardioneurological diseases (stroke, heart attack, aneurysms of the anterior communicating artery for 12 years without the repeated stroke catamnesis due to adequate predictive treatment, comatose states with consciousness renewal in non-perspective patients during half-year intensive treatment, epilepsy with stable residual period after 8–15 months of the vascular pathology correction, and autism with absolute socialization of the child and successful catamnesis at general school after 4 years of intensive purposeful vascular correction and multidisciplinary approach in neurorehabilitation (Figures 5, 6, 7, 8, 9, 10, 11, 12).

In the treatment process, it was succeeded to avoid critical moments due to purposeful medicinal cure of CVD patients under instrumental control. Patients in critical states (stroke, heart attack, comma, Epictetus, and apallic syndrome) during the rehabilitation course required permanent hemodynamic monitoring for the timely indication of pathological paradoxical reactions of the vascular system with the purpose of minimization of the state worsening and prevention of new crises.

An analysis of the acquired results shows that the organism of patients with CVS dysfunction in the process of the individually prescribed and controlled treatment has passed to the fundamentally new way—stable balance of hemodynamic processes in different CVS segments involving sanogenic autoregulated mechanisms of correction of adaptive CVS reconstructions. It appeared that capillary circulation, which carries out the basic function of the microcirculatory system (transcapillary exchange), *id est*, metabolism between blood and tissues, plays an important role in control of these processes. That is why periodic control of the microcirculation state in the process of treatment can be as an arbiter of well-being in the vascular macrosystem and in regional vascular reservoirs.

#### Recommendations

1. The major condition of overcoming the CVDs epidemics is a client-oriented (personalized) approach to every patient in particular that is based on principles of the evidence-based medicine. The logic of preventive approach to early diagnostics and treatment lays in deep knowledge of vascular pathology and laws of hemodynamics and analytical approaches to diagnostic potential of modern vascular technologies.

2. All investigations and monitoring of CVS functioning must be based on principles of evidence, informatively, specificity and high sensitiveness of modern technologies to disorders of various parameters of the vascular system in the process of pathological and sanogenic adaptive reconstructions in CVS provoked by a disease, processing different descriptions of vector of all segments in CVS local and regional levels with the specification on the damaged area and local influence of the area on the whole system functioning.

3. Any treatment course ultimately requires the CVS monitoring to analyze hemodynamic changes of adaptive or pathological reorganization in the vascular bed and the ability to predict any reactions of the disbalanced CVS to various internal and external stimuli.

### Predictive potential of the capillaroscopy for estimation of the vascular diseases' risk

Substantiating a problem of the human organism's senescence, Zalmanoff [35] considered that we should always observe the progressing decrease of the number of open capillaries, a part of them transformed into a state of shadows–non-functioning capillaries, for people of 40–45 years old [35]. This progressing drying out makes the anatomic–physiologic base of senescence. People of the elderly age have the velocity of blood flow that is reduced to one-third because of relative atony of capillaries in these people; their partial occlusion causes increasing resistance in the peripheral blood circulation. In the very senescence, capillaries become weaker, are winding, their diameter diminishes. Blood flow is slowing down. The above-stated data say for the indissoluble connection between heart functioning, chest volume, arterial discharge and venous inflow to the heart and, of course, between arterial and venous cerebral beds.

The term ‘capillary’ (from Latin *capillaris*, which means hair) combines the most thin-walled vessels of microcirculatory canal of size 8–12 μ. All human tissues are pierced by capillaries. As the capillary blood circulation makes the main function of the microcirculatory system (transcapillary exchange), that is metabolism between blood and tissues, condition of the microcirculation can be as an arbiter of welfare in the systemic hemodynamics and reflects the preserved arteriovenous balance in the regional vascular reservoirs.

Origin of any vascular pathology is associated primarily with the circulation disorders in the smallest blood vessels, the capillaries. Any reconstructions in the vascular system occur first in the microcirculatory level; therefore, monitoring of the capillary changes is the most informative in assessing the efficiency of a prescribed treatment [3,18,23,25,27,28].

*If the capillaries are evenly filled with blood, it is an evidence of the correct functioning of the system (heart → arteries → capillaries → veins → heart).* Thus, normal microcirculatory picture is an index of well-being in the whole cardiovascular system. That is why the capillaroscopy can be applied for screening early changes in vessels, and disorders in capillary picture require detailed examination of all segments in the cardiovascular system to detect and correct the disorders in the vascular ‘pipeline’.

A regular capillary has a hairpin form. Deviations from the ideal form indicate any pathology. Uneven blood supply, visualization of clots in the microcirculatory bed, or occurrence of the so-called capillary shadows signify disorders in blood pressure in the capillary or blood rheology.

Problems in the capillary functioning can cause circulatory disorders, leading to stagnation of the circulation and derangement of metabolic processes, and as a result, the immunity worsens, chronic diseases exacerbate, and new ones develop. Therefore, detection of any microcirculation problems at early stages is so important for forecasting the development of a cardiovascular disease and for effective treatment. For example, reduction of the circulation rate and formation of clots in microcirculatory bed can warn about the development of ischemic changes in the heart or in the brain and the threat of microthromboembolism.

Capillaroscopy is the one of the supplementary diagnostic methods of investigation that enables to observe the functioning of the peripheral section in the cardiovascular system of a human in the cutaneous and mucous surfaces. The technology is unique because it is created on the basis of new approaches—combination of technical components, new scientific knowledge of microcirculation, hemodynamics, and angioarchitectonics in a single diagnostic–rehabilitation complex. The capillaroscope is intended for an individual estimation of an adequate level of cortical blood supply according to necessities of cerebral tissues and for the examination of microcirculation condition.

Regular capillaroscopic examination allows for the diagnosis of microcirculation disturbances; control of non-invasively efficiency of prescription of an anti-aggregate therapy for patients with ischemic disease of heart, pancreatic diabetes, cancer neovascularization, etc.; and for studying the dynamics of microstructures' and materials' reactions. The capillaroscope is sensitive during examination in 98% of the cases (i.e., ischemic heart disease 100%, diabetes mellitus 50%, thrombi formation 80%, and abnormal vessel formations 100%). Our studies have showed that coma patients almost have no microcirculation in their finger nail beds. In dynamics, when blood supply increases for the brain and expressed signs of blood flow centralization are diminished, gradual blood flow is recommenced in the capillaries. This phenomenon can be as a prognostic criterion of positive changes in the treatment process. The capillaroscopic picture of stroke patients represents a tension degree of auto regulatory mechanisms at microcirculatory level and allows distinguishing basic pathogenetic links that require correction. Thus, capillaroscopy enables to choose the optimal approach to treatment scheme and to monitor its efficiency at individual evidence-based level.

Computer processing of the capillaroscopic investigations provides with the following:

• possibility of the *in vivo* visualization of microcirculatory changes in the capillary blood flow on a computer monitor screen;

• archiving of images of the capillary blood flow in a database and also review them in a free way;

strengthening of image contrasts;

• measurement of the capillary size, number of units of the regular blood elements;

• observation in dynamics of the capillary blood flow under the enlargements in 100 times; and

• prognosticating a pre-stroke and pre-infarction states of patients.

An advantage of smart capillaroscopy over other methods of microcirculation investigation is the visualization of the process, which significantly makes it easier for doctors to comprehend capillary pictures and enables them to study more deeply and to analyze the obtained data. Processing obtained images with the application of the mathematical modeling is another advantage because it enables us to analyze the picture of the microcirculation in more details and to present it in the figure characteristics that can add to the quantitative assessment of these virtual sections and reflect slight changes in the quantitative equivalent. A doctor needs only 5 min to get prior diagnosis of the presence or absence of any pathology in the cardiovascular system.

The device features, like easy to use, non-invasive, high quality of visualization, and archiving of static and video images, are very important for medical staff who use the smart capillaroscopy: mistaken diagnosis is practically excluded, results of the disease development are highly predictable, qualitative and quantitative assessment of the efficiency of the performed therapy can be done, and the possibility of obtaining unique microcirculatory changes for prognostication of sub- and decompensated states of patients.

Doctors' should pay attention to the necessity of the investigation and control of the dynamics of the size of the perivascular area to make objective assessment on the effect of diuretic therapy in patients with cardiac insufficiency and intracranial and intracellular hypertension.

The problem of the capillaroscopy application lies in the examination of microprocesses. Therefore, a diagnostic apparatus must be sensible enough, and instruments for measuring must be extraordinarily accurate. To avoid such errors, the software has been developed to hold accurate primary measuring and to get analytical conclusions, thanks to authorial algorithms put into basis of the data processing. The approach enables to get compared data and possibility to make correlation analysis between results of researches in different treatment periods.

#### Monitoring the treatment effectiveness with capillaroscopy

Using capillaroscopy, you can not only detect a disease but also periodically evaluate the treatment efficiency. The examination does not require any special preparation of the patient [9,25,27-29]. However, capillaroscopy application in dynamics with visualization of microcirculatory picture and data processing enables to make sure treatment efficiency, revealing tendencies in vascular reconstructions.

The clinic ‘Victoria Veritas’ has examined the efficiency of the vascular therapy by means of an innovative technology—dynamic capillaroscopy. The monitoring of the microcirculation processes starts during acute pharmacological tests. According to the clinic's data, the primary microcirculatory changes have appeared in 15–20th min of holding the acute pharmacological test in the form of reduction of expressiveness of the venular phlebectasia by 14%–29%, reduction of the perivascular edema area by 23%, and increase of velocity of pulse wave in capillaries in 73% of the examined patients.

After making the correlation analysis of the background data and conducting the treatment course, we have concluded using the capillaroscopy method that all patients had the positive dynamics of the microcirculatory picture. Positive changes took place mostly in the caliber and tonus of capillaries (in 67% of the examined patients), in a state of capillary filling (in 89% of the examined patients), and reduction of expressed capillary tortuosity (57% of patients) and size of the perivascular edema (96% of the examined patients who were diagnosed to have the edema during the background examination). There was a tendency to the arteriolar-venular balance (65% of patients) by the combined signs confirmed by accurate calculations.

#### ***The capillaroscopy complex***

The capillaroscopy complex includes the following

• technical device

• software

• references

• special training course for the device operation

supporting documents (certificates, licenses)

• information support.

**Figure 13:**
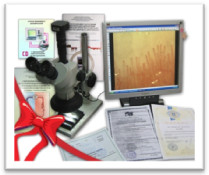
**The capillaroscopy complex.** For visualization, archiving, mathematical processing, and clinical interpretation of etiopathogenesis of microcirculatory disorders [28,30].

The device is demonstrated in Figure 13.

#### Recommendations

Regular capillaroscopic investigation enables to perform diagnostics of microcirculatory disorders, to control efficiency of the therapy almost for every patient non-invasively (psychoneurological, therapeutic, angiological, endocrinological, and cancer neovascularization profiles for the assessment of congenital and acquired vascular anomalies) and during prophylactic exanimations of healthy people who are under influence of external factors (stress, geomagnetic, temperature, and meteorological), and to control the state of railway men, pilots, and astronauts etc., who are connected with a risk due to their professions, resuscitation departments (control of a level of blood circulation restoration after critical states), insurance medicine (almost immediate determination of the cardiovascular system condition in the organism of an insurant), and sports medicine (control and dosage of the physical load system during sport trainings).

Thus, the described approaches to the analytical estimation of the vascular pipeline on macro- and microlevels allow newly considered problems in the blood supply for organs and systems in the human organism. Such innovative approach enables to estimate the possibility of sanogenic reconstructions in the vascular bed on local, regional, and systemic levels, and the adequacy of such alterations according to the necessities of the ill organism.

## Conclusions

Based on the data above, the following conclusions were made:

1. Integrative approach to overall examination of the vascular system requires permanent monitoring of the functional state of all segments of the cardiovascular system. Only predictive, preventive, and personalized CVS management enables to get stable results in those who struggle with CVD epidemic.

2. The proposed approach to diagnostics and treatment of disorders in functioning of the integral cardiovascular system has had long approbation by life; its efficiency has been proven by the evidence-based medicine methods and long-term catamnesis of crisis-free course of CVDs in treated patients [27].

3. Therefore, we offer Ukrainian vascular technologies as PPP approach for the correction of a vascular pathology (the technology for evidence-based vascular correction of pathological and sanogenic adaptive CVS reconstructions [25-28,30]. The technology enables not only to make accurate diagnoses but also to treat efficiently that reduces financial burden of a patient and his relatives. The presented medical technology will be useful in prophylactic and preventive medicine of the world for the health improvement of CVD patients.

4. The above-mentioned data show certain positive results in the approach to the examination and correction of cardiovascular diseases, but there are many unsolved problems in the vascular pathology field, which to our opinion, require joint efforts of the international scientific schools for a global scientific project.

## Consent

Written informed consent was obtained from the patients for publication of this report and for all accompanying images.

## Abbreviations

CVS: Cardiovascular system; CVDs: Cardiovascular diseases; PPP: Predictive, preventive and personalized.

## Competing interests

The authors declare that they have no competing interests.

## Authors’ contributions

UL is an initiator of the vascular angiopsychoneurology, proposed and developed principles for the arteriovenous balance in the human cardiovascular system and mathematical models of the hemodynamics in living systems, analyzed the efficiency of the applied technologies, and designed and drafted the manuscript. VN was engaged in modeling of biophysical processes in the human organism and modeling of biomechanics of the cardiovascular system. IB and NL carried out the diagnostics and treatments according to the proposed approaches. LR conducted the instrumental functional diagnostics and helped draft the manuscript. All authors read and approved the final manuscript.

## Authors’ information

UL is a doctor of medicine. She is an associate professor of the Nondestructive Control Department in Kyiv Polytechnic Institute, the head of the Department of Science, International Cooperation and Control on Medical Services in the Clinical Hospital ‘Feofania’, State Administration of the President’s Affairs of Ukraine. She is also an associate professor of the Medical Law Department in Solomon International University, Kyiv, Ukraine. In addition, she is an initiator of the vascular angiopsychoneurology. Furthermore, she is a member of the European Association of Neurologist and an academician of the European Academy of Natural Sciences. VN is a professor, a Ph.D. degree holder of Physical and Mathematical Sciences, an academician of the European Academy of Natural Sciences, a scientific consultant on biophysical problems in the scientific center ‘Veritas’, and the president of the social organization ‘Alcesta’. Also, he is the head of the Department of Analytical Mechanics and Control of Processes in Dynamic Systems in the Institute for Mathematics of National Academy of Sciences of Ukraine.
